# Prevalence of depression and anxiety among university students in Jeddah, Saudi Arabia: exploring sociodemographic and associated factors

**DOI:** 10.3389/fpubh.2024.1441695

**Published:** 2024-12-11

**Authors:** Azizah M. Malebari, Saeed O. Alamoudi, Talal I. AL-Alawi, Anas A. Alkhateeb, Adnan S. Albuqayli, Hamzah N. Alothmany

**Affiliations:** ^1^Department of Pharmaceutical Chemistry, Faculty of Pharmacy, King Abdulaziz University, Jeddah, Saudi Arabia; ^2^Faculty of Pharmacy, King Abdulaziz University, Jeddah, Saudi Arabia

**Keywords:** depression, anxiety, mental health, university students, student health, Saudi Arabia cross-sectional studies

## Abstract

**Introduction:**

Previous studies have shown that mental health issues such as depression and anxiety are on the rise globally, particularly among university students. The aim of this study is to assess the prevalence of depressive and anxiety symptoms among university students, and the associated potential risk factors, in Jeddah, Saudi Arabia.

**Methods:**

A cross-sectional questionnaire study was conducted in a sample of 728 students who anonymously completed three sets of questionnaires: a sociodemographic and lifestyle information questionnaire, the Patient Health Questionnaire-9 (PHQ-9) to screen for depressive symptoms and the Generalized Anxiety Disorder-7 (GAD-7) scale to screen for anxiety symptoms.

**Results:**

In a survey of 728 students, 81.5% reported depression and 63.6% anxiety, with no difference between medical and non-medical majors. Regular exercise, a history of psychological disorders, a diagnosis of chronic illness, and the use of antidepressant medications were significant indicators of depression. Significant anxiety markers included female gender, a diagnosis of chronic illness, a history of psychological disorders, the use of antidepressant medications, and smoking. Surprisingly, we did not observe any significant differences between the prevalence of depression or anxiety symptoms and common risk predictors, such as poor academic performance, low family income, and year of study.

**Conclusion:**

Depression and anxiety had a high prevalence among undergraduates, regardless of their field of study; therefore, we recommend the implementation and expansion of community-based mental health prevention programs and mentorship programs. Universities with counseling programs should identify and eliminate factors that contribute to depression and anxiety.

## Introduction

In recent years, the prevalence of depression and anxiety has increased, significantly affecting multiple aspects of personal and social well-being. There are several mental health challenges that can have detrimental effects on academic performance, decision-making, interpersonal relationships, and the overall quality of life of an individual (1). The lifetime prevalence rates of anxiety and major depression disorder are 12.1 and 16.6%, respectively (2). University students face particularly heightened risks due to factors such as academic stress, female gender, marital status, parental expectations, financial constraints, and high academic demands (3). These stressors can manifest in students as feelings of hopelessness, anger, lethargy, and distorted reality, and these depressive symptoms can result in mood fluctuations, aggressive behaviors, and substance abuse (3–5). Several studies have explored the prevalence of mental health problems among university students. For instance, at Franciscan University in Ohio, 33% of students reported experiencing depression and 40% reported anxiety (6). A Malaysian survey found that 37% of students had moderate stress, 63% had anxiety, and 23.7% had depression symptoms (7). In particular, medical students in the USA and Canada exhibited increased anxiety compared with the general population (8).

In the context of Saudi Arabia, 24.4 and 18% of King Faisal University students reported depression and anxiety, respectively, while 30% of Jazan University students experienced psychological disturbances (9, 10). According to Dammam University, Saudi Arabia, female medical students experienced lower wellbeing and higher anxiety during medical school than during their first year of bachelor’s studies (11).

Importantly, mental health challenges have academic repercussions; 41.2 to 44.3% of students reported feeling that their academic performance was hindered by emotional and mental issues, and 14.1 to 18.4% reported failing an academic requirement because of these challenges (12). While there is a growing body of research on mental health, a significant lack of understanding of mental health issues exists among university students in Jeddah, Saudi Arabia. Previously conducted studies have primarily focused on a variety of specific populations, including medical or nursing students, first-year students, or cohorts from specific institutions. As a result of this narrow focus, students from diverse backgrounds in the region cannot gain a comprehensive understanding of mental health challenges. Further, while numerous studies have examined depression and anxiety among students during the COVID-19 pandemic, our study was conducted in 2022, highlighting the need for ongoing evaluation of mental health post-pandemic (11, 13).

In recognition of the inadequate understanding of mental health issues among university students, this study aims to assess the prevalence of depression and anxiety among undergraduate students in Jeddah from various fields of study and academic years. This investigation aims to identify specific risk factors associated with mental health challenges within this population, highlighting the importance of continuing mental health support within academic institutions in order to promote the well-being of students throughout their educational journeys.

## Methods

### Study design and participants

The survey was conducted in September 2022 among university students in Jeddah, Kingdom of Saudi Arabia. Participants included university students in various fields.

### Sample size determination

The sample size was estimated to be 385 students, based on a sample size formula for cross-sectional study designs, using a margin of error of 5% and a confidence interval of 95%. This ensures that the findings are statistically reliable and representative of the population. Although the minimum sample size was determined to be 385, the study recruited 728 participants, which increases the statistical power and generalizability of the results.

### Data collection

The data were collected at the beginning of the semester (September 2022) before exams to eliminate the potential confounder of added exam-related stress. A link to the two-part Arabic/English self-administered surveys was posted on the official social networking sites for the university and its faculties (e.g., Whatsapp or Telegram) and on the university campus. In spite of the fact that the survey was made available online, efforts were made to minimize self-selection bias by targeting groups and departments uniformly. However, the possibility of self-selection bias remains a potential limitation since participation was voluntary. The first section of the survey concentrated on demographics. Specifically, this section gathered data on the participants’ age, gender, place of residence, field of study, academic level, academic year, marital status, living standard, grade point average (GPA), and family income. It also contained items related to lifestyle factors, including current smoking status and frequency of physical activity. Finally, this section included questions related to medical status, including the participant’s history of chronic conditions, history of mental conditions, and use of any medications for depression or anxiety.

The second section evaluated the students’ levels of anxiety and depression with the nine-item Patient Health Questionnaire-9 (PHQ-9) for depression symptoms and the seven-item Generalized Anxiety Disorder-7 (GAD-7) scale for anxiety symptoms.

The PHQ-9 assesses depressive symptoms, and Arabic and English versions were provided. The PHQ-9 is a valid and reliable tool for measuring symptoms of depression in all individuals, and the scores are divided into five categories: a score of <5 indicates no symptoms of depression, a score of 5 to 9 indicates mild symptoms, a score of 10 to 14 indicates moderate symptoms, a score of 15–19 indicates moderately severe symptoms, and of >20 indicates severe symptoms.

The GAD-7 is used in primary and outpatient settings to screen for generalized anxiety disorder. Arabic and English versions of the questionnaire were provided. The scores are classified into four categories: a score of <5 indicates no anxiety symptoms, a score of 5 to 9 indicates mild symptoms, a score of 10 to 14 indicates moderate symptoms, and a score of >15 indicates severe symptoms.

### Statistical analysis

Microsoft Excel 2016 (Microsoft® Corp., Redmond, WA, United States) was used for data entry, and the analyses were performed with IBM SPSS Statistics for Windows, version 26 (IBM Corp., Armonk, NY, United States). Descriptive statistics were used to summarize the data by estimating the frequency and percentage for categorical variables. Pearson’s Chi-squared test was used to identify significant differences in the prevalence of depression and anxiety between male and female students, with *p* < 0.05 indicating statistical significance.

## Results

### Sociodemographic characteristics

Table 1 provides the complete sociodemographic information for all participants. A total of 728 students participated in the study, with 55.63% males (*n* = 405) and 44.37% females (*n* = 323). Most participants were between 18 and 25 years old. The distribution of students across medical (45.74%, *n* = 333) and non-medical fields (54.26%, *n* = 395) was fairly even. First-year students made up the largest proportion of participants (27.47%, *n* = 200). A majority of students lived with their families (91.07%, *n* = 663) and were single (97.12%, *n* = 707). Regarding academic performance, 55.77% had a GPA above 4.25, while 44.23% had a GPA below this threshold. Family income was diverse, with 25.55% (*n* = 186) from low-income households and 13.19% (*n* = 96) from high-income households.

**Table 1:** Sociodemographic characteristics of the university student (*N* = 728).

Characteristics (*n* = 728)	Frequency (%)
Age	
18–25	700 (96.15)
26–35	28 (3.85)
Gender	
Male	405 (55.63)
Female	323 (44.37)
College specialty	
Medical colleges	333(45.74)
Non-medical colleges	395 (54.26)
Academic year	
First year	200 (27.47)
Second year	116 (15.93)
Third year	117 (16.10)
Fourth year	128 (17.58)
Fifth year	130 (17.86)
Sixth year	22 (2.83)
Seventh year	15 (2.16)
Place of living	
Family	663 (91.07)
Alone	37 (5.08)
College dorms	15 (2.06)
Friends	13 (1.79)
GPA	
5–4.75	168 (23.08)
4.74–4.25	238 (32.69)
4.24–3.75	185 (25.41)
3.74 and less	137 (18.82)
Marital status	
Single	707 (97.12)
Married	16 (2.20)
Widow	1 (0.14)
Divorced	4 (0.55)
Family income	
Low income (less than 5,000)	186 (25.55)
Middle low income (6,000–10,000)	116 (15.93)
Middle income (11,000–15,000)	168 (23.08)
Middle high income (16,000–24,000)	162 (22.25)
High income (more than 25,000)	96 (13.19)
Have a chronic illness	
Yes	72 (9.89)
No	656 (90.11)
Smoking	
Yes	144 (19.78)
No	584 (80.22)
Perform physical exercises	
Yes	192 (26.37)
No	536 (73.63)
Diagnosed previously with depression or anxiety	
Yes	124 (17.03)
No	604 (82.97)
Currently using antidepressant medications	
Yes	37 (5.08)
No	691 (94.92)

### Sociodemographic characteristics and levels of depression

**Figure 1:**
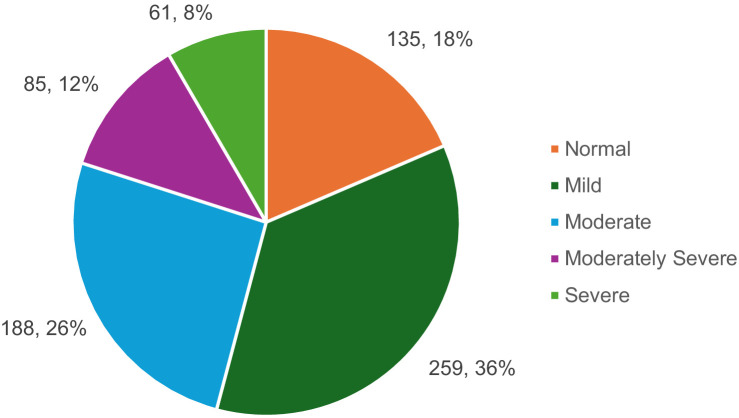
Depression prevalence among university students.

In this study, the pattern of depression prevalence differed from that of anxiety (Figure 1). According to the participants’ survey responses, 18.5% (*n* = 135) did not have depression, 35.6% (*n* = 259) had mild depression, 25.8% (*n* = 188) had moderate depression, 11.7% (*n* = 85) had moderately severe depression, and 8.4% (*n* = 61) had severe depression. Depression prevalence varied across different demographic groups (Table 2). Among students aged 18–25, 16.8% had no symptoms, 35.4% had mild depression, and 26% had moderate depression, while 11.7 and 8.3% exhibited moderately severe and severe depression, respectively. A similar trend was seen among single students, with 18.2% showing no depression and 8.3% experiencing severe depression. First-year students showed a more balanced distribution, with 20.5% reporting no depression and 10.5% having severe depression. Students living with their families had a lower prevalence of severe depression (8.0%), while those with a GPA between 4.25 and 4.74 showed resilience, with 20.2% having no symptoms of depression. Students from low-income families showed lower rates of severe depression, with 18.3% reporting no symptoms, while students with chronic illnesses exhibited higher rates of severe depression (13.9%).

**Table 2:** Sociodemographic characteristics and different level of depression.

		Depression				
Sociodemographic characteristics	None	Mild	Moderate	Moderately severe	Severe	*p*-value
Age (in years)						
18–25	130 (16.8)	248 (35.4)	182 (26)	82 (11.7)	58 (8.3)	
26–35	5 (17.9)	11 (39.3)	6 (21.4)	3 (10.7)	3 (10.7)	0.968
Total	135 (18.5)	259 (35.6)	188 (25.8)	85 (11.7)	61 (8.4)	
Gender						
Male	77 (19)	156 (38.5)	105 (25.9)	39 (9.6)	28 (6.9)	
Female	58 (18)	103 (31.9)	83 (25.7)	46 (14.2)	33 (10.2)	0.094
Total	135 (18.5)	259 (35.6)	188 (25.8)	85 (11.7)	61 (8.4)	
Material status						
Single	129 (18.2)	254 (35.9)	184 (26)	81 (11.5)	59 (8.3)	
Married	5 (31.3)	4 (25)	3 (18.8)	4 (25)	0 (0)	
Divorced	1 (25)	1 (25)	0 (0)	0 (0)	2 (50)	0.09
Widow	0 (0)	0 (0)	1 (100)	0 (0)	0 (0)	
Total	135 (18.5)	259 (35.6)	188 (25.8)	85 (11.7)	61 (8.4)	
Year of study						
First year	41 (20.5)	61 (30.5)	54 (27)	23 (11.5)	21 (10.5)	
Second year	17 (14.7)	40 (34.5)	34 (29.3)	18 (15.5)	7 (6)	
Third year	17 (14.5)	40 (34.2)	30 (25.6)	19 (16.2)	11 (9.4)	
Fourth year	27 (21.1)	49 (38.3)	27 (21.1)	14 (10.9)	11 (8.6)	0.585
Fifth year	27 (20.8)	50 (38.5)	33 (25.4)	10 (7.7)	10 (7.7)	
Sixth year	4 (18.2)	11 (50)	6 (27.3)	0 (0)	1 (4.5)	
Seventh year	2 (13.3)	8 (53.3)	4 (26.7)	1 (6.7)	0 (0)	
Total	135 (18.5)	259 (35.6)	188 (25.8)	85 (11.7)	61 (8.4)	
Place of living						
Family	127 (19.2)	231 (34.8)	173 (26.1)	79 (11.9)	53 (8)	
Alone	3 (8.1)	18 (48.6)	6 (16.2)	4 (10.8)	6 (16.2)	
College dorms	1 (6.7)	5 (33.3)	7 (46.7)	1 (6.7)	1 (6.7)	0.283
Friends	4 (30.8)	5 (38.5)	2 (15.4)	1 (7.7)	1 (7.7)	
Total	135 (18.5)	259 (35.6)	188 (25.8)	85 (11.7)	61 (8.4)	
GPA						
5–4.75	37 (22)	67 (39.9)	38 (22.6)	14 (8.3)	12 (7.1)	
4.74–4.25	48 (20.2)	89 (37.4)	53 (22.3)	27 (11.3)	21 (8.8)	
4.24–3.75	30 (16.2)	64 (34.6)	50 (27)	29 (15.7)	12 (6.5)	0.085
3.74 and less	20 (14.6)	39 (28.5)	47 (34.3)	15 (10.9)	16 (11.7)	
Total	135 (18.5)	259 (35.6)	188 (25.8)	85 (11.7)	61 (8.4)	
Family income						
Low income (less than 5,000)	34 (18.3)	67 (36)	47 (25.3)	21 (11.3)	17 (9.1)	
Middle low income (6,000–10,000)	22 (19)	44 (37.9)	28 (24.1)	13 (11.2)	9 (7.8)	
Middle income (11,000–15,000)	39 (23.2)	46 (27.4)	48 (28.6)	24 (14.3)	11 (6.5)	
Middle high income (16,000–24,000)	25 (15.4)	64 (39.5)	43 (26.5)	17 (10.5)	13 (8)	0.738
High income (more than 25,000)	15 (15.6)	38 (39.6)	22 (22.9)	10 (10.4)	11 (11.5)	
Total	135 (18.5)	259 (35.6)	188 (25.8)	85 (11.7)	61 (8.4)	
Have chronic illness						
Yes	6 (8.3)	20 (27.8)	21 (29.2)	15 (20.8)	10 (13.9)	
No	129 (19.7)	239 (36.4)	167 (25.5)	70 (10.7)	51 (7.8)	**0.005**
Total	135 (18.5)	259 (35.6)	188 (25.8)	85 (11.7)	61 (8.4)	
Smoking						
Yes	20 (13.9)	51 (35.4)	39 (27.1)	21 (14.6)	13 (9)	
No	115 (19.7)	208 (35.6)	149 (25.5)	64 (11)	48 (8.2)	0.463
Total	135 (18.5)	259 (35.6)	188 (25.8)	85 (11.7)	61 (8.4)	
Perform physical exercises						
Yes	43 (22.4)	81 (42.2)	41 (21.4)	15 (7.8)	12 (6.3)	
No	92 (17.2)	178 (33.2)	147 (27.4)	70 (13.1)	49 (9.1)	0.017
Total	135 (18.5)	259 (35.6)	188 (25.8)	85 (11.7)	61 (8.4)	
Diagnosed with depression or anxiety						
Yes	2 (1.6)	21 (16.9)	37 (29.8)	30 (24.2)	34 (27.4)	
No	133 (22)	238 (39.4)	151 (25)	55 (9.1)	27 (4.5)	**0.0001**
Total	135 (18.5)	259 (35.6)	188 (25.8)	85 (11.7)	61 (8.4)	
Used antidepressants medications						
Yes	0 (0)	10 (27)	12 (32.4)	4 (10.8)	11 (29.7)	
No	135 (19.6)	249 (36)	176 (25.5)	81 (11.7)	50 (7.2)	**0.0001**
Total	135 (18.5)	259 (35.6)	188 (25.8)	85 (11.7)	61 (8.4)	

The significant *p*-values are denoted in bold.

### Sociodemographic characteristics and different levels of anxiety

**Figure 2:**
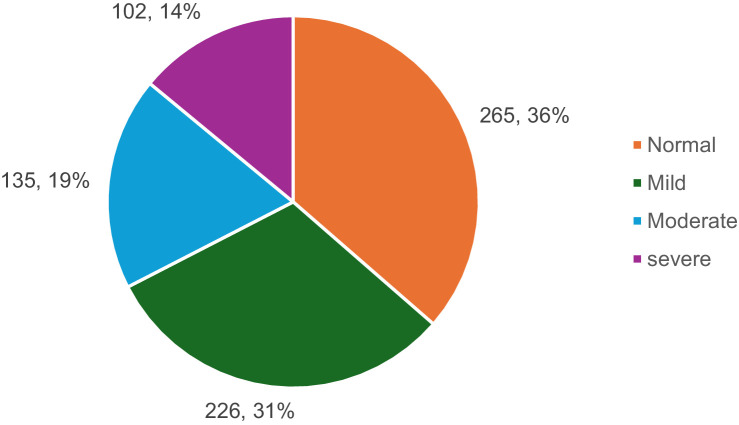
Anxiety prevalence among university students.

As shown in Figure 2 36.4% of participants (*n* = 265) did not have anxiety, 31.0% (*n* = 226) had mild anxiety, 18.5% (*n* = 135) had moderate anxiety, and 14.0% (*n* = 102) had severe anxiety. The relationship between sociodemographic factors and anxiety is shown in Table 3. Among students aged 18–25, 36.6% reported no anxiety, while 31.0% had mild anxiety, 18.3% had moderate anxiety, and 14.1% had severe anxiety. Female students experienced higher levels of anxiety than males, with 19.8% of females reporting severe anxiety compared to 9.4% of males. First-year students exhibited a similar pattern, with 17.5% experiencing severe anxiety. Students living with family had lower anxiety levels overall, with 35.9% reporting no anxiety and only 14.5% experiencing severe anxiety. Academic performance also played a role; students with a GPA between 4.25 and 4.74 showed lower levels of anxiety, with 40.8% reporting no anxiety. Among students from low-income families, 33.3% reported no anxiety.

**Table 3:** Sociodemographic characteristics and different level of anxiety.

		Anxiety			
Sociodemographic characteristics	None	Mild	Moderate	Severe	*p*-value
Age (in years)					
18–25	256 (36.6)	217 (31)	128 (18.3)	99 (14.1)	
26–35	9 (32.1)	9 (32.1)	7 (25)	3 (10.7)	0.792
Total	265 (36.4)	226 (31)	135 (18.5)	102 (14)	
Gender					
Male	162 (40)	138 (34.1)	67 (16.5)	38 (9.4)	
Female	103 (31.9)	88 (27.2)	68 (21.1)	64 (19.8)	**0.0001**
Total	265 (36.4)	226 (31)	135 (18.5)	102 (14)	
Marital status					
Single	259 (36.6)	215 (30.4)	135 (19.1)	98 (13.9)	
Married	5 (31.3)	9 (56.3)	0 (0)	2 (12.5)	
Divorced	1 (25)	1 (25)	0 (0)	2 (50)	0.143
Widow	0 (0)	1 (100)	0 (0)	0 (0)	
Total	265 (36.4)	226 (31)	135 (18.5)	102 (14)	
Year of study					
First year	77 (38.5)	52 (26)	36 (18)	35 (17.5)	
Second year	41 (35.3)	37 (31.9)	22 (19)	16 (13.8)	
Third year	31 (26.5)	36 (30.8)	32 (27.4)	18 (15.4)	
Fourth year	48 (37.5)	42 (32.8)	20 (15.6)	18 (14.1)	0.269
Fifth year	52 (40)	45 (34.6)	19 (14.6)	14 (10.8)	
Sixth year	10 (45.5)	7 (31.8)	4 (18.2)	1 (4.5)	
Seventh year	6 (40)	7 (46.7)	2 (13.3)	0 (0)	
Total	265 (36.4)	226 (31)	135 (18.5)	102 (14)	
Place of living					
Family	238 (35.9)	206 (31.1)	123 (18.6)	96 (14.5)	
Alone	14 (37.8)	12 (32.4)	8 (21.6)	3 (8.1)	
College dorms	5 (33.3)	5 (33.3)	3 (20)	2 (13.3)	0.825
Friends	8 (61.5)	3 (23.1)	1 (7.7)	1 (7.7)	
Total	265 (36.4)	226 (31)	135 (18.5)	102 (14)	
GPA					
5–4.75	59 (35.1)	57 (33.9)	27 (16.1)	25 (14.9)	
4.74–4.25	97 (40.8)	63 (26.5)	41 (17.2)	37 (15.5)	
4.24–3.75	64 (34.6)	63 (26.5)	37 (20)	21 (11.4)	0.554
3.74 and less	45 (32.8)	43 (31.4)	30 (21.9)	19 (13.9)	
Total	265 (36.4)	226 (31)	135 (18.5)	102 (14)	
Family income					
Low income (less than 5,000)	62 (33.3)	69 (37.1)	27 (14.5)	28 (15.1)	
Middle low income (6,000–10,000)	47 (40.5)	36 (31)	21 (18.1)	12 (10.3)	
Middle income (11,000–15,000)	71 (42.3)	36 (21.4)	39 (23.2)	22 (13.1)	
Middle high income (16,000–24,000)	50 (30.9)	55 (34)	33 (20.4)	24 (14.8)	0.12
High income (more than 25,000)	35 (36.5)	30 (31.3)	15 (15.6)	16 (16.7)	
Total	265 (36.4)	226 (31)	135 (18.5)	102 (14)	
Have chronic illnesses					
Yes	18 (25)	17 (23.6)	15 (20.8)	22 (30.6)	
No	247 (37.7)	209 (31.9)	120 (18.3)	80 (12.2)	**0.0001**
Total	265 (36.4)	226 (31)	135 (18.5)	102 (14)	
Smoking					
Yes	38 (26.4)	53 (36.8)	29 (20.1)	24 (16.7)	
No	227 (38.9)	173 (29.6)	106 (18.2)	78 (13.4)	0.046
Total	265 (36.4)	226 (31)	135 (18.5)	102 (14)	
Perform physical exercises					
Yes	80 (41.7)	62 (32.3)	27 (14.1)	23 (12)	
No	185 (34.5)	164 (30.6)	108 (20.1)	79 (14.7)	0.127
Total	265 (36.4)	226 (31)	135 (18.5)	102 (14)	
Diagnosed with depression or anxiety					
Yes	10 (8.1)	27 (21.8)	37 (29.8)	50 (40.3)	
No	255 (42.2)	199 (32.9)	98 (16.2)	52 (8.6)	**0.0001**
Total	265 (36.4)	226 (31)	135 (18.5)	102 (14)	
Used antidepressants medications					
Yes	4 (10.8)	11 (29.7)	6 (16.2)	16 (43.2)	
No	261 (37.8)	215 (31.1)	129 (18.7)	86 (12.4)	**0.0001**
Total	265 (36.4)	226 (31)	135 (18.5)	102 (14)	

The significant *p*-values are denoted in bold.

### Lifestyle factors and mental health outcomes

In total, 9.89% of participants (*n* = 72) reported having a chronic illness, 19.78% (*n* = 144) were smokers, and 26.37% (*n* = 192) engaged in regular physical exercise. Of the students, 17.03% (*n* = 124) had a previous diagnosis of depression or anxiety, and 5.08% (*n* = 37) were currently taking antidepressants (Table 1).

Among smokers, the prevalence of mild to severe depression was higher compared to non-smokers, while 22.4% of those who exercised regularly reported no symptoms of depression. Students with a history of psychological disorders displayed a higher prevalence of moderate to severe depression, and those on antidepressant medications had higher depression severity (Table 2).

For anxiety, a wide range of severity levels was reported among smokers, while 41.7% of students who exercised regularly reported no symptoms of anxiety. Students with a history of psychological disorders had a higher prevalence of severe anxiety (40.3%). Among students taking anxiety medications, 43.2% experienced severe anxiety (Table 3).

### Prevalence of anxiety among male and female students

Table 4 highlights the differences in anxiety prevalence between male and female students. Among smokers, male students were more likely to report normal anxiety levels (22.2%) than female students (4.2%), while female smokers were more likely to report severe anxiety (11.1%) compared to males (5.6%) (*p* < 0.001).

**Table 4:** Sociodemographic prevalence of anxiety among male and female students.

	Normal	Mild	Moderate	Severe	*p*-value
Smoking					
Male	32 (22.2)	43 (29.9)	21 (14.6)	8 (5.6)	
Female	6 (4.2)	10 (6.9)	8 (5.6)	16 (11.1)	**0.0001**
Total	38 (26.4)	53 (36.8)	29 (20.1)	24 (16.7)	
Have a chronic illness					
Male	13 (18.1)	11 (15.3)	7 (9.7)	9 (12.5)	
Female	5 (6.9)	6 (8.3)	8 (11.1)	13 (18.1)	**0.0001**
Total	18 (25.0)	17 (23.6)	15 (20.8)	22 (30.6)	
Diagnosed with depression or anxiety					
Male	2 (1.6)	15 (12.1)	15 (12.1)	19 (15.3)	
Female	8 (6.5)	12 (9.7)	22 (17.7)	31 (25.0)	**0.0001**
Total	10 (8.1)	27 (21.8)	37 (29.8)	50 (40.3)	
Used antidepressants medication					
Male	1 (2.7)	5 (13.5)	4 (10.8)	5 (13.5)	
Female	3 (8.1)	6 (16.2)	2 (5.4)	11 (29.7)	**0.0001**
Total	4 (10.8)	11 (29.7)	6 (16.2)	16 (43.2)	

The significant p-values are denoted in bold.

Among students with chronic illnesses, 18.1% of males reported normal anxiety levels compared to 6.9% of females, while females exhibited a higher prevalence of severe anxiety (18.1% vs. 12.5% in males) (*p* < 0.001). Similarly, among students taking antidepressants, 29.7% of females experienced severe anxiety compared to 13.5% of males (*p* < 0.001).

## Discussion

To the best of our knowledge, this is the first comprehensive analysis of the prevalence of symptoms of depression and anxiety, as well as their associated factors, among college students in Jeddah, Saudi Arabia. Although the PHQ-9 and GAD-7 scales are not diagnostic tools, they are valuable for identifying the prevalence and severity of depression and anxiety symptoms in this demographic.

In recent years, the prevalence of psychological disorders, notably anxiety and depression, has increased among university students. Such disorders not only impact academic success but also affect students’ well-being. This study examined epidemiological determinants and their correlation with mental health outcomes, particularly depression and anxiety, among college students in Jeddah, Saudi Arabia. A sample size of 728 participants allowed for an in-depth analysis of depression and anxiety and their correlated demographic and lifestyle factors. We found a prevalence of depression and anxiety of 81.5 and 63.6%, respectively, significantly higher than those reported in previous Saudi studies (9, 10).

The COVID-19 pandemic significantly influenced the epidemiology of mental health disorders (14, 15). Disruptions in healthcare delivery systems compromised patients’ ability to consult healthcare providers and adhere to medication schedules. Additionally, changes necessitated by the pandemic, such as reduced physical activity, increased social isolation, dietary changes, and the shift to remote learning, contributed to heightened rates of psychological disorders. The widespread sense of fear and uncertainty surrounding the pandemic was exacerbated by confinement measures, amplifying the incidence of anxiety and depression in the general population, including university students (16–18).

Our investigation highlighted the impact of four key categories—gender-specific patterns, academic performance, health-related factors, and socio-demographic influences—on the prevalence of depression and anxiety among students.

### Gender-specific prevalence of depression and anxiety

Our findings reveal that gender disparities in anxiety levels among university students are pronounced and complex. Our data on the sociodemographic factors of this cohort support earlier research suggesting that gender is significantly associated with mental health outcomes (19). Specifically, although our study found no significant differences in depression rates between genders, females demonstrated a notably higher prevalence of anxiety than males. These results contrast with the findings of studies in Jazan and Al-Hasa, which showed that females were more susceptible to both depression and anxiety (5, 20). However, our observations are similar to those of a Malaysian study, which found no significant gender difference in depression but did identify a marked difference in anxiety levels between males and females (21). Furthermore, another study indicated that the anxiety rate in females was twice the rate in males (22). This gender-based difference in anxiety levels is also consistent with the findings of a recent study at Qassim University (23), which reported a similar general trend of a higher prevalence of anxiety in female Saudi students. The intricate interplay of female hormones, especially the fluctuations of progesterone and estrogen during various menstrual phases, might contribute significantly to the heightened prevalence of anxiety disorders in females (19).

### Academic factors affecting depression and anxiety

In the academic category, we analyzed the effect of GPA and academic year. While these factors may strongly influence students’ future career prospects, our results did not reveal significant associations between these variables and the prevalence of anxiety or depression. This finding contradicts the results in the existing literature; one previous study reported that anxiety levels varied according to academic standing (4), whereas others have found an inverse relationship between GPA and anxiety (5, 24) and increased depressive symptoms in students with higher GPAs. Furthermore, one study found that students with higher scores tended to have more symptoms of depression (20). A significant contributor to this discrepancy may be the cultural context within Saudi Arabia, in which academic success may not be subjected to the same level of pressures as in other educational settings. The strong family and community support systems in our cohort may mitigate the perceived stress associated with academic achievement. During the COVID-19 pandemic, the transition to remote learning may have altered the landscape of academic pressures, affecting the perception of students’ performance.

Medical students face significant challenges in their education that can impact their mental health; these challenges are often attributed to the intense curriculum and high academic workload (25–27). Studies comparing the prevalence of mental disorders between medical and non-medical students have shown varied results. A British study reported lower depression rates among medical students (28), whereas a Swedish counterpart reported a higher prevalence of depression in medical students (29).

According to our investigation, there was no statistically significant difference between medical and nonmedical students in terms of anxiety and depression rates. According to a study conducted at Umm Al-Qura University in Makkah, Saudi Arabia, no significant differences were found in depression prevalence between these groups, but anxiety levels were elevated among nonmedical students (30). By contrast, a study on Saudi Arabian students reported higher rates of both anxiety and depression in medical students than in their nonmedical peers (11, 31). Similarly, another study involving Portuguese students found elevated rates of these conditions in medical students than in nonmedical students (32). Notably, among Saudi medical students, males showed higher burnout rates, whereas females, especially those raising children, faced an increased risk of depression due to emotional exhaustion and cynicism (33).

### Health-related factors and their influence on depression and anxiety

Health-related factors were also notable, specifically the influence of smoking, physical activity, and medication use. Our analysis illustrated significant differences in the prevalence of depression and anxiety based on health-related variables, albeit with exceptions for smoking and physical activity. This aligns with prior studies that have reported a significant link between poor physical health, medication misuse, and depression (4, 34, 35). Moreover, our data on health factors are similar to those in the existing literature, with the exception of smoking (15). A number of demographic factors influenced anxiety prevalence, revealing clear gender differences. For instance, 22.2% of male smokers had normal anxiety levels, compared to 4.2% of female smokers (*p* < 0.001). Moreover, 11.1% of female smokers displayed severe anxiety compared with 5.6% of males (*p* < 0.001). This pattern of smoking and anxiety supports previous findings that females who smoke tend to experience higher levels of stress and anxiety (36). While many studies have established a strong association between smoking and psychological disorders, our results indicate that this association is less pronounced (37). Increasing health awareness and changing attitudes about smoking could influence self-reported behaviors and mental health.

Regarding the influence of chronic illnesses on anxiety levels, our findings highlight the gender disparity reported in a previous study, which reported that females with chronic health conditions were more prone to heightened anxiety levels (34). Chronic diseases significantly influence the prevalence of depression and anxiety. Cardiovascular disease; COPD; cancer; chronic pain conditions, such as fibromyalgia and arthritis; and neurological conditions, such as multiple sclerosis and Parkinson’s, are associated with increased rates of these mental health issues. It is vital to address the mental health of patients with these chronic illnesses. In addition, gender may influence the psychological consequences of chronic diseases (38). Furthermore, the trend we observed in which females diagnosed with psychological disorders exhibited a higher prevalence of depression than males aligns with previous findings suggesting that a history of psychiatric disorders significantly influences the prevalence of depression (30). According to our findings, the mental well-being of university students in Jeddah requires significant attention. It is crucial to establish relevant and robust mental health services tailored to this population’s needs.

### Socio-demographic influences on depression and anxiety

Regarding personal factors, including financial status and residential situation, we observed no notable differences in the prevalence of depression or anxiety. These results corroborate previous findings asserting no significant association between personal factors and mental health (5). Conversely, several studies have reported a significant correlation between these personal metrics and mental disorders (4, 34, 39). Discrepancies between our findings and existing literature may be attributed to cultural, methodological, or sample differences. Access to mental health resources and financial context in our urban cohort were likely to have an impact on outcomes. The majority of public universities charge no tuition, and students receive government allowances, live with their families, and have minimal financial obligations, reducing stress associated with education.

## Limitations

Self-reporting depression and anxiety symptoms has several drawbacks, including social desirability bias to escape negative judgment; thus, students may have underreported their symptoms. Furthermore, many people are unable to recognize their depression or anxiety, possibly because they cannot articulate their feelings. This may have led to erroneous severity evaluations and underreporting of symptoms. In addition, if individuals have inadequate self-awareness, they might not be fully aware of their emotional states and could struggle to analyze their symptoms appropriately. This may have resulted in either an under- or overreporting of symptoms. Furthermore, because this study was cross-sectional, we cannot conclude whether sociodemographic factors, such as smoking, chronic diseases, and physical exercise, are causes or risk factors of depression or anxiety.

## Recommendations

Overall, even when applying very conservative diagnostic criteria, self-reported depression or anxiety symptoms should not be equated to the clinical diagnosis of depression or anxiety. However, longitudinal studies that follow cohorts of students throughout their education could elucidate the development and contributing factors of mental distress among students.

## Conclusion

Depression and anxiety are among the most common conditions in primary care, and they are highly prevalent in undergraduate students regardless of their field of study. These disorders are often called invisible diseases because it can be very difficult to identify and diagnose them, especially in students. In this study, gender emerged as a key determinant, with females displaying a consistently higher prevalence of anxiety than their male counterparts. This trend has also been observed by many studies conducted locally and globally. Lifestyle factors (e.g., smoking habits) and health-related concerns, especially a history of chronic diseases and psychiatric disorders, were significantly associated with anxiety and depression levels. It is recommended that universities implement concrete interventions to support student mental health based on these findings. An integrated approach to mental health services and academic advising is recommended, as well as stress-reduction programs and targeted mental health resources for female students. A community-based mental health prevention program and mentorship program can also enhance support systems for students, ultimately fostering a healthier academic environment.

## Data Availability

The raw data supporting the conclusions of this article will be made available by the authors, without undue reservation.
